# A UHV MOKE magnetometer complementing XMCD-PEEM at the Elettra Synchrotron

**DOI:** 10.1107/S1600577521002885

**Published:** 2021-03-30

**Authors:** Francesca Genuzio, Tomasz Giela, Matteo Lucian, Tevfik Onur Menteş, Carlo Alberto Brondin, Giuseppe Cautero, Piotr Mazalski, Stefano Bonetti, Jozef Korecki, Andrea Locatelli

**Affiliations:** a CERIC-ERIC, Basovizza, Trieste, Italy; b National Synchrotron Radiation Centre SOLARIS, Jagiellonian University, Kraków, Poland; c Elettra–Sincrotrone Trieste SCpA, Basovizza, Trieste, Italy; d Department of Molecular Sciences and Nanosytems, Ca’ Foscari University of Venice, Venezia, Italy; e Faculty of Physics, University of Białystok, Białystok, Poland; f Jerzy Haber Institute of Catalysis and Surface Chemistry, Polish Academy of Sciences, Kraków, Poland; g Department of Physics, Stockholm University, Stockholm, Sweden

**Keywords:** Magneto-Optical Kerr Effect, MOKE magnetometry, XMCD-PEEM, SPELEEM, *in situ* studies

## Abstract

We report on a custom-built UHV-compatible Magneto-Optical Kerr Effect (MOKE) magnetometer for applications in surface and materials sciences, operating in tandem with the PhotoEmission Electron Microscope (PEEM) endstation at the Nanospectroscopy beamline of the Elettra synchrotron. The magnetometer features a liquid-nitrogen-cooled electromagnet that is fully compatible with UHV operation and produces magnetic fields up to about 140 mT at the sample. Longitudinal and polar MOKE measurement geometries are realized. The magneto-optical detection is based on polarization analysis using a photoelastic modulator. The sample manipulation system is fully compatible with that of the PEEM, making it possible to exchange samples with the beamline endstation, where complementary X-ray imaging and spectroscopy techniques are available. The magnetometer performance is illustrated by experiments on cobalt ultra-thin films, demonstrating close to monolayer sensitivity. The advantages of combining *in situ* growth, X-ray Magnetic Circular Dichroism imaging (XMCD-PEEM) and MOKE magnetometry into a versatile multitechnique facility are highlighted.

## Abstract 2

A UHV-compatible MOKE magnetometer for *in situ* studies operating in tandem with the PEEM at the Nanospectroscopy beamline of the Elettra synchrotron.

## 1. Introduction   

The downscaling of magnetic structures, driven by the need for ever smaller and efficient magnetic storage, has stimulated intense research in systems with film thicknesses limited to a few atomic layers and a lateral size well below the micron level (Vaz *et al.*, 2008 ▸). As applications demand increasingly smaller devices, fabrication and characterization techniques face similar challenges, making it crucial to develop versatile measurement facilities with high magnetic sensitivity and lateral resolution (Sander *et al.*, 2017 ▸). Nowadays, a wide range of experimental techniques is available for the magnetic characterization of a variety of micro- and nanostructures, exploiting the interaction of photons or electrons with matter, or using a magnetic material as probe (Bland & Mills, 2006 ▸). At synchrotrons, X-ray magnetic dichroism has gained prominence, owing to its elemental specificity, capability to probe spin and orbital moments, and high sensitivity in detecting signals from ultra-thin films and dilute magnetic systems (Stöhr, 1995 ▸). The combination of X-ray Magnetic Circular Dichroism with X-ray PhotoEmission Electron Microscopy (XMCD-PEEM) (Bauer, 2014 ▸; Feng & Scholl, 2019 ▸) or Scanning Transmission X-ray Microscopy (STXM) (Stoll *et al.*, 2015 ▸) has fostered great progress in the field of ultra-thin-film magnetism. In particular, there has been a plethora of studies using XMCD-PEEM imaging on magnetic domains, domain walls, their transformations and dynamics (Cheng & Keavney, 2012 ▸). PEEM provides lateral resolution close to the nm level, as well as a time resolution below 100 ps (Feng & Scholl, 2019 ▸). On the other hand, the presence of high voltages in the sample environment, the sensitivity of electron beams to applied magnetic fields, the probing depth limited to a few nanometres and the extra effort needed to obtain a full vectorial magnetization map all constitute challenges in using XPEEM as a stand-alone complete magnetic characterization technique. In order to overcome these limitations, a multitechnique experimental approach is therefore highly desirable.

Magneto-Optical Kerr Effect (MOKE) magnetometry is one of the most important techniques for magnetic characterization under external fields. Magneto-optical effects involve a modification of the light polarization upon interaction of the photon beam with a magnetic material and can be detected by performing a polarization analysis of the scattered light (Kerr, 1877 ▸; Zak *et al.*, 1990 ▸). The choice of the scattering geometry allows the magnetization along different directions to be probed. Nowadays, MOKE is typically performed using a photoelastic element, also known as a photoelastic modulator (PEM) modulating the polarization of the photon beam. This method was pioneered by Sato (1981 ▸, 1993 ▸), who demonstrated the simultaneous measurement of Kerr rotation and reflectance magneto-circular dichroism. The theoretical framework for quantitative evaluation of the data, as well as the efficiency of the apparatus, is well established (Polisetty *et al.*, 2008 ▸). At the state of the art, the polarization modulation technique allows three-dimensional (3D) quantitative mapping of the magnetization vector (Vavassori, 2000 ▸). Microscopic imaging applications using the Kerr effect have also been a prolific approach in magnetism research, with micrometre lateral resolution due to the diffraction limit at visible wavelengths (Freeman *et al.*, 1998 ▸; Ishibashi *et al.*, 2006 ▸; Srivastava *et al.*, 2018 ▸). Most notably, surface MOKE enables the sensitivity to a single atomic layer to be reached, which makes it highly suitable for the study of ultra-thin magnetic films (Bader *et al.*, 1986 ▸; Qiu & Bader, 2000 ▸; Usov *et al.*, 2005 ▸). Ultra-high vacuum (UHV) operation is also feasible, although hampered by space constraints and the difficulty of operating strong electromagnets in a vacuum. Nonetheless, the literature reports various examples of custom-built UHV MOKE set-ups, which combine very-high magnetic sensitivity and integration with other magnetic probes (Peterka *et al.*, 2003 ▸; Lehnert *et al.*, 2009 ▸; Kumar *et al.*, 2008 ▸; Vinai *et al.*, 2020 ▸).

We describe here a MOKE magnetometer that has been developed to work in tandem with the Spectroscopic PhotoEmission and Low-Energy Electron Microscope (SPELEEM III, Elmitec GmbH) installed at the Nanospectroscopy beamline at the Elettra synchrotron (Locatelli *et al.*, 2006 ▸; Menteş *et al.*, 2014 ▸). Thus, the laterally resolved chemical, structural and magnetic information provided by the X-ray PEEM microscope can be integrated with the behaviour under field, obtained by MOKE. By enabling measurements in both longitudinal and polar geometries, our MOKE opens the possibility of drawing a full vectorial mapping of the magnetization, an invaluable help in understanding canted configurations than cannot be resolved by XMCD-PEEM only.

Along with the UHV environment necessary for ultra-thin-film studies, our MOKE set-up features polarization modulation by means of PEM, in order to maximize magnetic sensitivity. To the best of our knowledge, this is the only MOKE installation that is equipped with a sample stage compatible with the SPELEEM and a UHV sample-transfer section, herewith referred to as a *vacuum bag*, to transport samples between the two set-ups. In the following, we present the vacuum chamber, optical layout and data acquisition system of the newly developed MOKE apparatus. Subsequently, the magnetometer performance is demonstrated by recording the in-plane and out-of-plane hysteresis loops of ultra-thin Co films, down to a thickness of approximately 1 nm.

## 2. System description   

As underlined in the *Introduction* [Sec sec1], the MOKE magnetometer (MM) hereafter described has been conceived as a magnetic characterization tool complementary to the SPELEEM microscope at the Nanospectroscopy beamline of Elettra. Its operation requires full compatibility of the sample manipulation and transfer system used at the beamline endstation. For this purpose, the MOKE set-up has been designed to accommodate the SPELEEM sample cartridge and other custom-developed cartridges of similar type (Foerster *et al.*, 2016 ▸). The key elements of the apparatus, namely the UHV operation, the optical set-up and the acquisition software, are described in the following.

### 2.1. Vacuum chamber layout   

**Figure 1:**
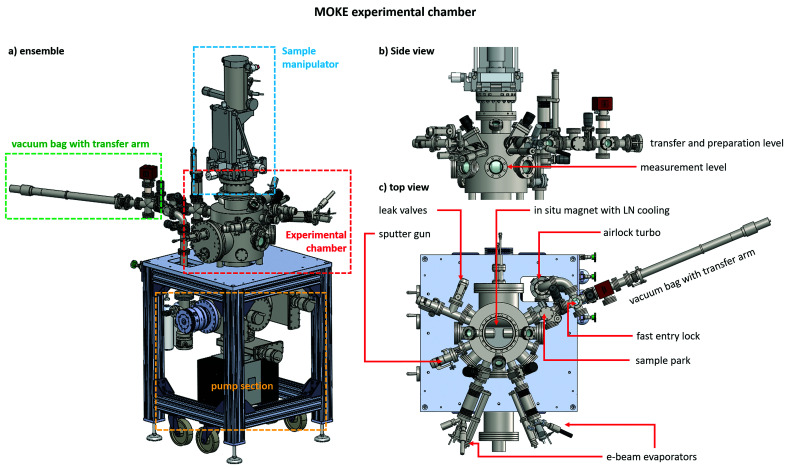
(*a*) 3D view of the UHV MOKE system. The dashed boxes identify the principal parts. (*b*) Side view of the experimental chamber, which is split over two levels, namely, (i) transfer and preparation, and (ii) measurement. (*c*) Top view of the experimental chamber.

A 3D drawing of the MM apparatus is shown in Fig. 1 ▸(*a*). Labels and dashed boxes indicate the principal elements: manipulator, experimental chamber, vacuum bag with transfer arm and pumping stage. The vacuum pumps are positioned in the lower part of the vacuum vessel, under the chassis supporting the vacuum chamber. In order to efficiently pump the vacuum vessel, three different pumps are used, all mounted on DN160CF flanges in order to maximize pumping speed: an ion pump (Varian Starcell 300), a Non Evaporable Getter (NEG) pump (SAES getters C500-MK2-ST707) and a turbo pump (Agilent TwisTorr 305 FS). Gate valves (Allectra 515-GV-C160) are installed at the inlet flange of each pump, permitting the individual isolation of the pumps from the vacuum vessel. This is particularly useful to protect the NEG and ion getter from exposure to ambient conditions during maintenance operations. The turbo pump is typically used during bake-out, as well as when performing chemical treatments in a vacuum. The ion and NEG pumps enable vibration-free UHV operation and result in a very efficient pumping speed of residual gases, hydrogen and hydrocarbons in particular. The base pressure of the chamber is 7 × 10^−11^ mbar.

The experimental chamber is spread over two levels, as can be seen in Fig. 1 ▸(*b*). The upper level is dedicated to sample preparation and transfer, whereas the MOKE measurements are carried out at the lower level. The height difference between the two levels, 120 mm, has been kept as small as possible in order to minimize the overall length of the manipulator and thus the vibrations on the sample during measurements. The top view of the set-up (Fig. 1 ▸ *c*) illustrates the geometrical arrangement of the side flanges. The upper part of the chamber is equipped with three viewports that facilitate sample manipulation and transfer operations.

The other CF38 flanges host the equipment for sample preparation. Two e-beam (PREVAC EBV 40 A1) evaporators are available for the deposition of metallic films. An effusion-cell (Tecnoproject SRL) enabling the deposition of organic layers can be mounted in place of one of the e-beam evaporators. A sputter gun (PREVAC IS 40 C1) permits sample cleaning by Ar-ion bombardment. Leak valves are installed for dosing gases (H_2_, O_2_, hydrocarbons, *etc*.) during chemical treatments. A sample parking stage can store up to four sample cartridges in UHV. A gate valve (VAT) separates the main UHV chamber from the fast entry lock, which is pumped by a dedicated turbo pump (Pfeiffer Hipace 80). This is backed by a dry pump (Vacuubrand MV 2 N T), using an independent vacuum line.

In the lower level of the experimental chamber, there are five DN63CF flanges equipped with viewports at 45° intervals, their axes pointing to the centre of the vessel (Figs. 1 ▸ *b* and 1 ▸ *c*). As detailed in the next section, this solution enables MOKE measurements to be performed either in the polar or in the longitudinal geometry, using different arrangements of the optical set-up. Longitudinal MOKE is carried out at an angle of incidence of about 45° on the sample. This value is dictated by the large diameter of the poles of the magnet, ∼50 mm, which accommodate the ‘bulky’ sample cartridge used in the SPELEEM. The size of the sample cartridge imposes, in fact, a rather large gap between the poles of the magnet (40 mm).

The electromagnet, built at the AGH University of Science and Technology in Krakow, is fully compatible with *in situ* UHV operation. The magnetic core and poles have been realized using ARMCO steel. To reach the desired magnetic properties, all parts were baked in hydrogen at 950 °C for 10 h, followed by a slow cooling to room temperature (8 h). The coil surrounding the core is contained in a sealed vacuum-tight stainless-steel enclosure, inside which liquid nitrogen (LN_2_) is circulated to provide cooling.

**Figure 2:**
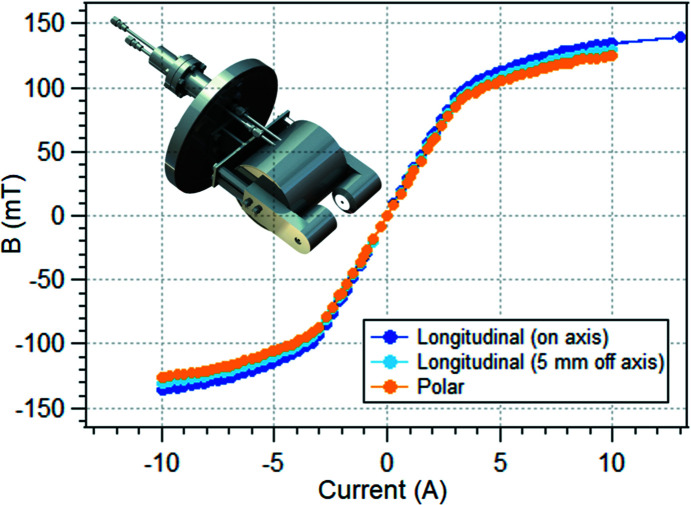
Magnetic field *versus* applied current at different sample positions between the poles: on-axis polar (2 mm from the pole surface), on-axis longitudinal (at the middle of the poles) and off-axis longitudinal (at the middle of the poles and 5 mm off the central axis). The inset shows a 3D drawing of the electromagnet.

A 3D drawing of the electromagnet is shown in the inset of Fig. 2 ▸. A small through-hole (diameter of 5 mm) has been drilled along the axis of the magnetic poles, permitting the laser beam to reach the sample in polar MOKE geometry (we note that the size of the laser beam is typically below 1 mm at the hole entrance). The design of the poles was optimized using finite element analysis, with the aim of maximizing the uniformity of the magnetic field at the measurement position.

A KEPCO BOP36-28MG power supply is used to provide the coil with electrical current. To avoid overheating at high currents, the coil must be operated cold. Under these conditions, a maximum current of 13 A can be applied, creating a field of about 140 mT at the sample. Room-temperature operation is allowed up to a maximum current of 4.0 A. To avoid artifacts determined by heating of the coil, the KEPCO supply is always operated in current control mode. Plots of the magnetic field *versus* applied current are shown in Fig. 2 ▸ for the polar and longitudinal measurement configurations. Note that the field strength is only weakly dependent on the exact sample position within the region between the magnetic poles.

The sample manipulator was custom designed and produced by PREVAC. It allows *xyz* movement (*x*, *y*: ±25 mm; *z* motion: 150 mm) and ±180° rotation, for precise sample positioning and orientation. Electrical connections to the sample cartridge permit the sample to be heated and its temperature monitored. A dedicated power supply allows the delivery of up to 2.6 A to the cartridge filament for radiative heating; electron bombardment heating is also possible, applying a voltage bias between the sample and the filament. Electron bombardment heating is typically employed to perform prolonged thermal treatment up to 1200 K, or brief flashes up to 2000 K. The sample temperature is measured by a W/Re thermocouple (type C). The manipulator also allows LN_2_ cooling. During cooling, the cold finger temperature was checked with a chromel/alumel thermocouple. It takes about 60 min to reach a temperature of −120 °C from room temperature, and about 2 h to reach the minimum temperature of −135 °C. The manipulator also hosts a quartz crystal microbalance, in order to precisely tune the deposition rate of the e-beam evaporators pointing at the sample.

The MM is also equipped with a vacuum bag (VB), which can be easily detached from the chamber and connected to the SPELEEM microscope operating at the Nanospectroscopy beamline. The vacuum bag is separated from the fast entry lock by a DN38CF gate valve (VAT). The VB hosts a small support for the sample and a transfer arm, which combines linear and rotary motion and allows the sample to be moved to and from the experimental chamber, the airlock or the VB. UHV conditions in the VB are preserved using a hybrid pump (SAES Getters NEXTORR Z 200), which combines ion and NEG technologies. The base pressure in the VB is 3 × 10^−11^ mbar.

### 2.2. Optical layout   

The standard light source of the set-up is a stabilized HeNe laser with an output power of 1.2 mW and a wavelength close to 633 nm (Thorlabs HRS015B). This laser can maintain intensity and frequency stability over time (0.01° and ±2 MHz for 1 h, respectively). Another laser source, with a wavelength of 405 nm, is also available, in order to access a wider range of materials (*e.g.* perovskites) with optimal performance. Two detectors, a Hinds Instruments DET 200-002 and a DET 200-004, are available, optimized for the different frequencies of the two laser sources.

**Figure 3:**
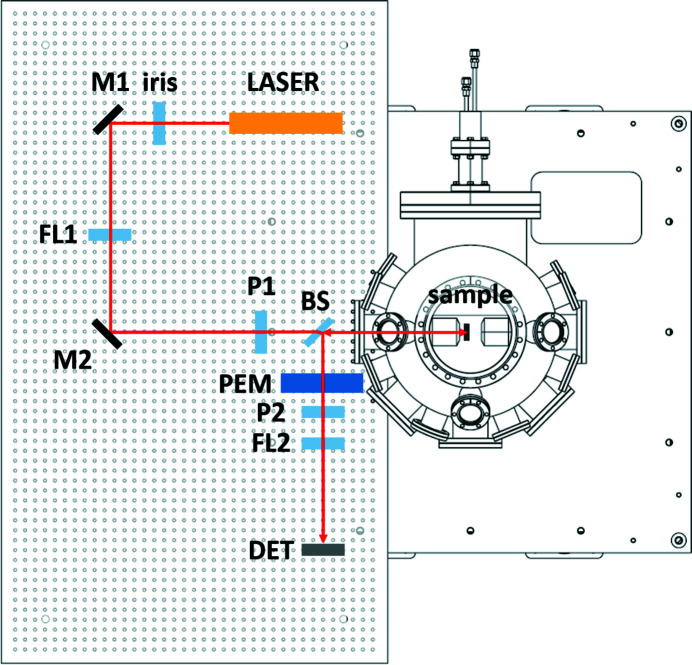
Optical layout for the polar MOKE measurement configuration. Labels indicate the main optical elements: alignment mirrors (M1 and M2), focusing lenses (FL1 and FL2), beam splitter (BS), polarizers (P1 and P2), photoeleastic modulator (PEM) and detector (DET). One or more irises can be used during alignment.

The optical set-up allows either the polar or the longitudinal measurement geometry to be realised, as illustrated in Figs. 3 ▸ and 4 ▸, respectively. In both configurations, the optical table, a custom-designed aluminium-made breadboard (Thorlabs), is fixed tightly on the chassis sustaining the vacuum chamber.

**Figure 4:**
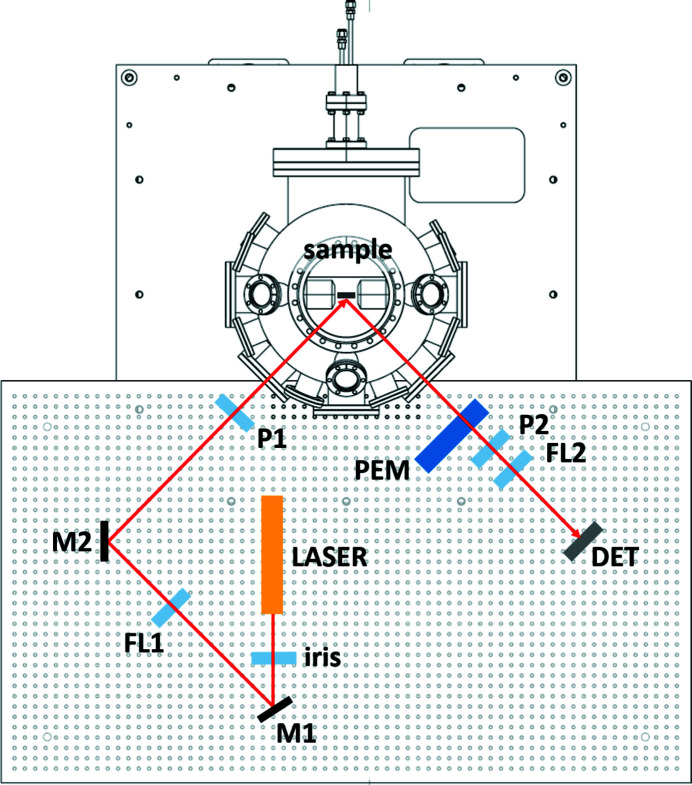
Optical layout for the longitudinal MOKE measurement configuration. Labels indicate the optical elements.

In the polar set-up (Fig. 3 ▸), the laser beam impinges on the sample at normal incidence, passing through a small circular aperture in one of the poles of the magnet. The mirrors M1 and M2 facilitate laser alignment, and the lens FL1 permits a precise focusing of the laser light on the sample. After reflections on the sample and the beam splitter, the beam passes through the photoelastic modulator (PEM) (Hinds Instruments PEM I/FS-50) and finally impinges on detector D, focused by FL2. In the longitudinal MOKE geometry (Fig. 4 ▸), the laser beam impinges on the sample at about a 45° incidence angle. In this measurement configuration, no beam splitter is needed in order to separate the incoming and reflected beams.

In both the polar and longitudinal set-ups, two Glan–Taylor polarizers (Thorlabs GT10-A), labelled P1 and P2 in Figs. 3 ▸ and 4 ▸, are inserted in the optical path, mounted on high-precision rotation mounts (Thorlabs PRM1GL10/M). P1 defines the linear polarization of the incoming laser beam; the analyzer P2, noncollinear to P1, defines a new polarization axis. In this manner, the magnetic signal corresponding to the polarization rotation is filtered out from the non-magnetic Compton scattering, making it possible to detect the tiny intensity changes induced by the sample magnetization. We could verify that the best signal-to-noise ratio is typically obtained close to extinction, in agreement with the literature (Buchner *et al.*, 2016 ▸; Allwood *et al.*, 2003 ▸). Nonetheless, quantitative measurements of the Kerr rotation have been performed by setting the relative angle between P1 and P2 at 45°.

Since the rotation caused by a few atomic layers of magnetic material is of the order of a few millidegrees, the detector output is typically processed with modulation techniques (Sato, 1981 ▸). In our case, we chose a commercial PEM from Hinds Instruments. This device modulates the polarization of the laser beam at high frequency (*f* = 50 kHz), permitting signal filtering and amplification by means of a signal conditioning unit (Hinds Instruments SCU-100) and lock-in amplifier (Hinds Instruments Signaloc Model 2100).

## 3. Data acquisition and processing   

**Figure 5:**
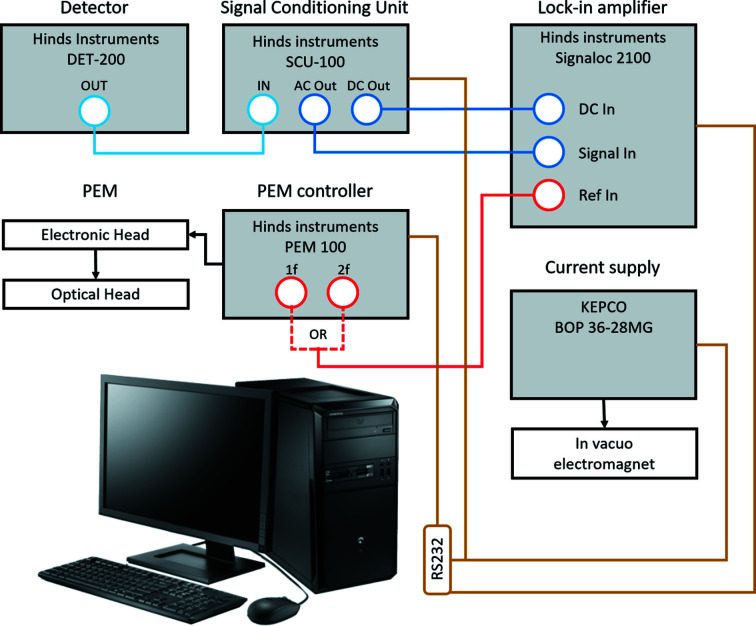
Scheme of the MOKE electronics and data acquisition measurement set-up.

The data acquisition set-up is illustrated in Fig. 5 ▸. The PEM optical head modulates the polarization of the laser beam at a frequency *f* = 50 kHz, driven by a dedicated controller (Hinds Instruments PEM-100). The frequency-modu­lated laser beam is focused onto the detector, generating an electrical signal that exhibits the same frequency modu­lation as the optical signal. The detector output is then processed by the signal conditioning unit (Hinds Instruments SCU-100), which separates out and amplifies with selectable gains the AC and DC signal components. The lock-in amplifier (Hinds Instruments Signaloc 2100) then extracts the AC component at frequency *f* or 2*f*, tuning the bandpass filter using the reference signal provided by the PEM controller. The lock-in delivers two outputs, proportional to amplitude of the DC and AC components, respectively, which are finally read by the data acquisition PC *via* the RS232 port.

**Figure 6:**
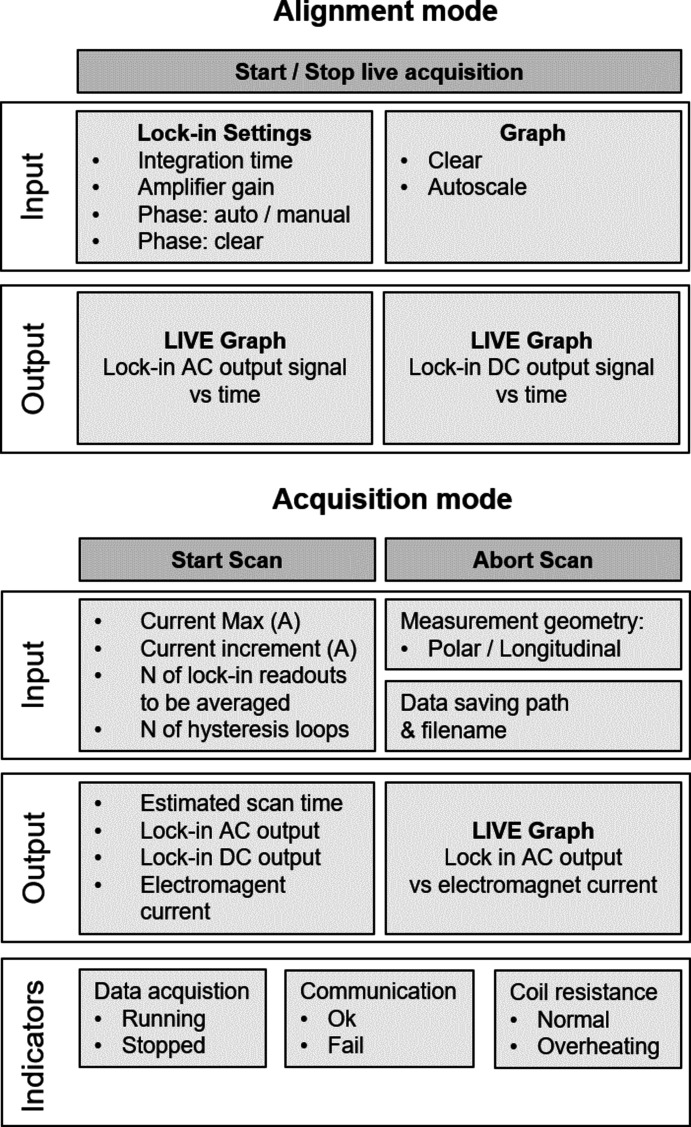
Diagram illustrating the two main operation modes of the MOKE control software: alignment and acquisition. The main functionalities are presented, together with input and output.

The MOKE control software has been written using *LabVIEW*. A diagram illustrating the main functionalities of the software is shown in Fig. 6 ▸. The end-user can select two main operating modes, namely, alignment and data acquisition. In the alignment mode, the program produces live plots of the lock-in DC and AC output signals, which greatly facilitate the optimization of the magnetometer optical alignment. In the data acquisition mode, the graphical user interface (GUI) allows the operator to input all relevant parameters of the MOKE scan, including the range and increment of the electromagnet current and the number of consecutive hysteresis cycles to be performed. The program also allows the lock-in acquisition settings to be controlled, *e.g.* the time constant of the input filter of the amplifier (4–516 ms) and averaging, frequency (1*f* or 2*f*, with *f* = 50 kHz) and amplification (0–15 dB). Based on the selected input parameters, the code estimates the scan duration, providing the operator with a feedback for planning the data acquisition.

During data collection, the program drives the electromagnet, ramping the current up or down in steps while performing a hysteresis loop. At each step, the program waits for the current to be stabilized. Then, the computer reads the DC and AC lock-in output signals, averaging multiple read-outs into a single data point. The resulting signal is plotted *versus* the magnetic field, which is calculated using a calibration table taking into account the chosen measurement geometry. The acquisition continues until the planned hysteresis loops are completed, or the scan is aborted. The AC and DC raw intensity data are saved in an ASCII file along with the electromagnet current values.

The resistance of the coil is monitored constantly during the scans. The acquisition is automatically interrupted when the resistance exceeds a given threshold, indicating coil overheating. The communication between the acquisition software and the various instruments are monitored constantly. In case of failure, the software resets the communication, restarting the data collection after correct functioning is restored.

Each MOKE data set is saved as a separate ASCII file. It can be loaded for display using a custom-made software suite that is run under the graphing and data analysis program *IGOR Pro* (Wavemetrics Inc., https://www.wavemetrics.com/). This software allows us to load and plot individual hysteresis loops and their average. After loading the data, the mean value of the AC signal is subtracted from each loop before calculating the averaged loop. For hysteresis loops recorded at 45°, the Kerr rotation angle, θ_K_, is calculated as follows (Oakberg, 2019 ▸):
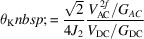
Here, 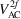 is the amplitude of the 2*f* AC signal component (measured tuning the lock-in amplifier to 2*f*) and *V*_DC_ is the amplitude of the corresponding DC signal. Both the AC and the DC signal amplitudes are normalized to the respective gains of the signal conditioning unit, *G*_AC_ and *G*_DC_. *J*_2_ = 0.4318 is the value of second-order Bessel function at a retardation of 2.405 rad, which ensures that the DC component becomes independent of θ_K_. In practice, a calibration of the PEM retardation settings is necessary to operate under this condition. In this work, the hysteresis loops measured with the second polarimeter close to extinction were rescaled to the Kerr rotation value obtained at 45°.

The graphing software also converts the current provided to the electromagnet to field values in units of mT. The field value for a given current is calculated using a cubic spline interpolation between the points of the current–field data shown in Fig. 2 ▸.

## 4. Application examples   

### 4.1. Spin reorientation transition in Ga^+^-irradiated Co–Pt heterostacks   

Ion irradiation provides a well-known method to tune the magnetic anisotropy in thin films, enabling also lithographic applications (Chappert *et al.*, 1998 ▸). In Al_2_O_3_/Pt 20 nm/Co 3.3 nm/Pt 5 nm, Ga^+^ irradiation is known to induce a spin reorientation transition (SRT) within the magnetically active Co layer from an in-plane to an out-of-plane direction. As previously shown, the SRT in Pt/Co/Pt multilayers is precisely controlled *via* the ion fluence (Maziewski *et al.*, 2012 ▸, 2015 ▸). Since MOKE hysteresis loops readily reveal changes in the magnetic anisotropy, selected Pt/Co/Pt samples with varying ion fluences were used to demonstrate and test longitudinal and polar MOKE operations during the commissioning of the MOKE apparatus.

The samples were grown *ex situ* at the Institute of Physics of the Polish Academy of Science in Warsaw, using molecular beam epitaxy. The as-grown films (Co thickness of 3.3 nm) exhibit in-plane magnetic anisotropy. Ion irradiation was performed at the Helmholtz Zentrum Dresden–Rossendorf in Dresden, exposing the entire sample area to 30 keV Ga^+^ ions (Maziewski *et al.*, 2012 ▸). The samples were subsequently measured with the MOKE magnetometer at Elettra.

**Figure 7:**
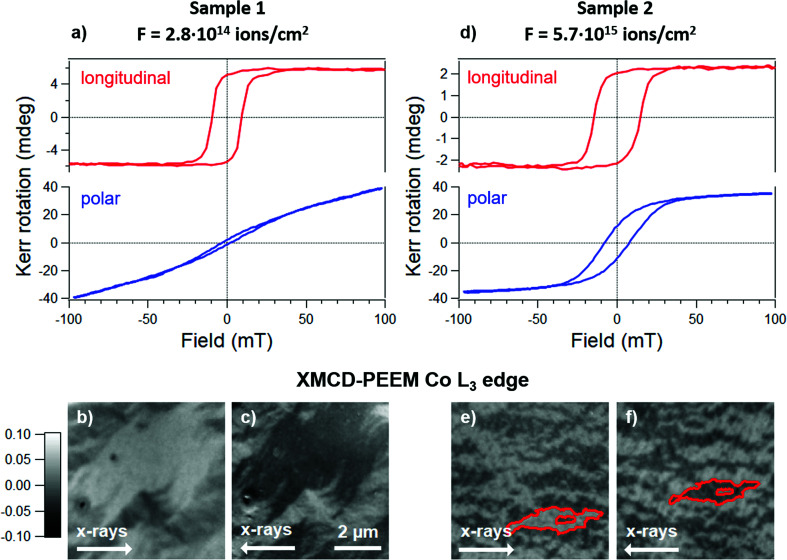
(*a*, *d*) Longitudinal and polar MOKE loops (see labels) of Pt/Co/Pt test samples irradiated with fluence (**1**) *F* = 2.8 × 10^14^ ions cm^−2^ and (**2**) *F* = 5.7 × 10^15^ ions cm^−2^. For both samples, the measurements were carried out at 3° from extinction using the following acquisition parameters: AC gain = 5× [10× for the longitudinal loop in part (*a*)], DC gain = 1×, PEM frequency *f* = 50.037 kHz and lock-in amplifier tuned on 2*f*. Each data set is the average of five loops; 160 (320) data points were acquired for longitudinal (polar) scans. The overall acquisition time was 339 s (678 s). (*b*, *c*; *e*, *f*) Co *L*_3_ edge (*h* = 778.1 eV) XMCD-PEEM images of the same samples at room temperature after demagnetization by a slowly decreasing oscillating magnetic field oriented along the out-of-plane direction. The XMCD asymmetry is indicated on the left. The same specimen region is depicted before and after sample rotation by 180° with respect to the direction of the photon beam. The inversion of contrast demonstrates in-plane magnetic anisotropy. The red contour helps visualization of the same domain.

Fig. 7 ▸ displays the MOKE longitudinal and polar hysteresis loops of samples that were exposed to different ion fluences, along with with XMCD-PEEM images of the demagnetized state at room temperature. In part (*a*), the sample was irradiated at very low fluence (*F* = 2.8 × 10^14^ ions cm^−2^). The relatively sharp square loop observed in longitudinal geometry, together with the almost linear field-dependence measured in polar geometry, suggest in-plane easy axis orientation. This is confirmed by the quantitative analysis of the corresponding loops, showing a larger coercivity (9.3 ± 0.15 mT) in the loop measured in longitudinal geometry compared with the value of 4.5 ± 0.3 mT for the polar loop.

To corroborate the picture of in-plane magnetization, we performed XMCD-PEEM on the same sample. Note that, owing to the grazing incidence of the photon beam on the sample (16°), the SPELEEM offers high sensitivity to the in-plane magnetization component. Prior to the experiments, the sample was demagnetized in the out-of-plane direction, favouring the formation of large in-plane domains. The XMCD-PEEM images in Figs. 7 ▸(*b*) and 7 ▸(*c*) show the same sample region after rotation of the sample manipulator by 180°. Due to the measurement geometry, the XMCD contrast of in-plane domains is inverted by a sample rotation of 180°. Conversely, the XMCD contrast of the out-of-plane domains is invariant to any azimuthal rotation. Thus, the observed inversion of contrast arises from the predominant in-plane nature of the magnetic domains.

At higher ion fluences (*F* = 5.7 × 10^15^ ions cm^−2^), the MOKE loops are significantly different (see Fig. 7 ▸ *d*). The longitudinal MOKE loop also shows a square shape with slightly increased coercivity, Yet, the Kerr rotation at remanence is more than halved with respect to the less irradiated sample (2.09 ± 0.02 mdeg instead of 5.22 ± 0.05 mdeg), suggesting a decrease of the net magnetization in the in-plane direction. Further, an evident deviation from the linear regime is observed in polar geometry, revealing the opening of a hysteresis loop in the direction normal to the surface plane. The Kerr rotation at remanence, determined for the polar loops after subtracting the linear paramagnetic contribution, changes notably, increasing from 1.7 ± 0.2 to 14.5 ± 0.1 mdeg. At the same time, the coercivity nearly doubles, increasing from a value of 4.5 ± 0.3 to 7.9 ± 0.1 mT, which is still lower than the value measured in longitudinal geometry (14.3 ± 0.1 mT).

The above values indicate that, at a fluence of 5.7 × 10^15^ ions cm^−2^, the magnetization has some tendency to tilt towards the out-of-plane direction. As can be seen in the XMCD-PEEM image in Fig. 7 ▸(*e*), the morphology of the magnetic domains is changed with respect to that observed in the first sample. The relatively strong image contrast asymmetry of about 10% suggests that the magnetization still exhibits in-plane characteristics. This is confirmed by the inversion of contrast upon sample rotation by 180°. The development of a domain pattern with smaller size, as well as observation of XMCD contrast after saturation along the in-plane direction (not shown), however, suggests the development of an out-of-plane component. Apparently, at this fluence, the critical point of the in-plane to out-of-plane SRT is being approached.

### 4.2. Magnetism in graphene/Co/Re(0001)   

Graphene–cobalt ultra-thin heterostructures are attracting increasing scientific attention due to the strong perpendicular magnetic anisotropy (PMA) that graphene induces in cobalt (Yang *et al.*, 2016 ▸). Further interest arises from the fact that, when cobalt is deposited on a heavy-metal support, peculiar spin textures are observed, with the out-of-plane magnetic domains sep­arated by chiral Néel domain walls (Chen *et al.*, 2015*a* ▸,*b* ▸; Boulle *et al.*, 2016 ▸). This observation hints that magnetic skyrmions can be nucleated and mani­pulated in suitably engineered thin films, leading to a practical realization of the racetrack memory (Parkin *et al.*, 2008 ▸).

With this motivation, we recently investigated the magnetic properties of ultra-thin Co on Re(0001), focusing our attention on the effect of carbon adsorption on the film magnetic anisotropy. Our previous work had established a means to graft the magnetic state by accumulating carbon adspecies on the cobalt surface using e-beam-stimulated CO fragmentation (Genuzio *et al.*, 2019*a* ▸; Genoni *et al.*, 2018 ▸). Interestingly, we found that these species can be converted to a graphitic overlayer upon performing a moderate thermal treatment, without compromising the integrity of the ultra-thin Co film. Combined photoemission spectroscopy and XMCD-PEEM measurements demonstrated that the cobalt underneath the graphene exhibits enhanced PMA (Genoni *et al.*, 2018 ▸).

Graphene/Co/Re(0001) provides us with an intriguing model system to carry out test experiments using the MM. Owing to the very low Co thickness, in the range 4.5 to 4.7 AL (about 1 nm), the experiments demand very high magnetic sensitivity. The availability of PEEM is key during preparation, as well as for microscopic characterization with multiple techniques. During experiments, several sample transfers between MOKE and PEEM are necessary. In this respect, the *vacuum bag* is essential for avoiding sample exposure to the atmosphere and preserving intact the magnetic properties of the as-grown film in UHV.

The graphene/Co(4.7 AL)/Re(0001) sample was prepared following the cleaning and Co deposition protocols described in our previous studies (Genoni *et al.*, 2018 ▸; Genuzio *et al.*, 2019*a* ▸). The graphene layer was grown using chemical vapour deposition (CVD) of ethylene at a partial pressure in the range from 1 × 10^−7^ to 1 × 10^−6^ mbar, saturating the Co surface at room temperature and then annealing it up to about 700 K for a total time of at least 30 min (Jugovac *et al.*, 2019 ▸). A small amount of oxygen (*P*[O_2_] = 2 × 10^−8^ mbar) was introduced during the initial stage of graphene growth in order to lower the nucleation density and increase the crystalline quality of the film.

The crystallographic and structural quality of the film was checked *in situ* using the multi-technique approach of the SPELEEM. XPS was used to probe the carbon and oxygen signals after growth. Namely, the C 1*s* core level emission was found to exhibit a sharp line profile centred at 284.9 eV, typical of graphitic carbon. No traces of oxygen could be detected. LEEM imaging at high resolution proved the lateral homogeneity of the graphene film. No indications of dewetting and growth of 3D Co crystals were found. Microspot Low Energy Electron Diffraction (LEED) provided evidence that the graphene overlayer is rotationally incoherent, as suggested by the development of a corona with a radius corresponding to the reciprocal lattice vectors of graphene.

**Figure 8:**
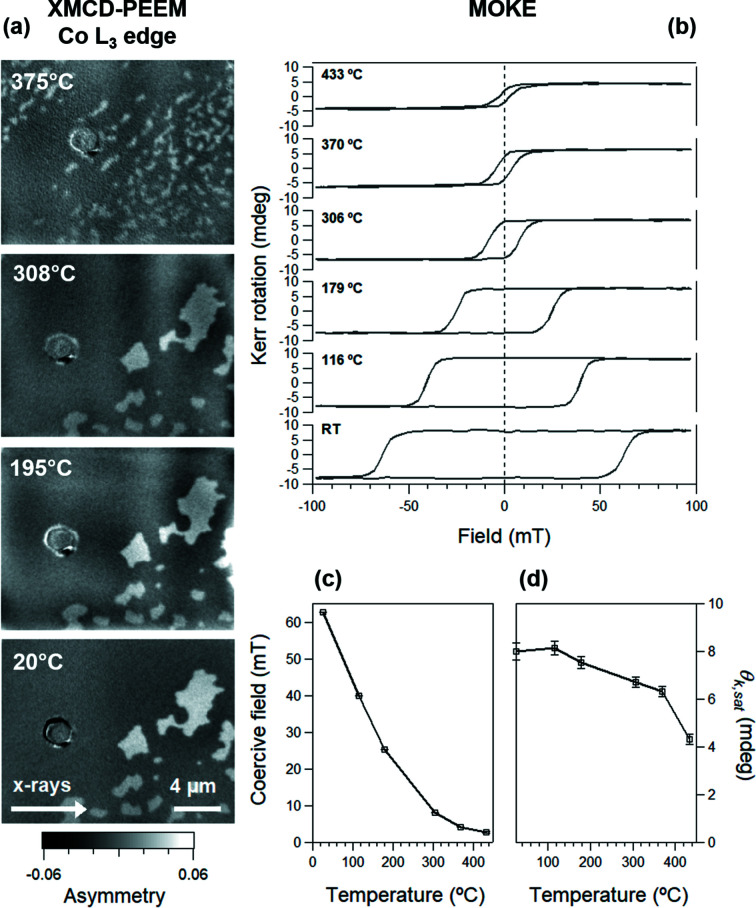
(*a*) XMCD-PEEM at the Co *L*_3_ edge, showing the out of-plane magnetic domains in Gr/Co/Re; from left to right: room temperature, 195, 308 and 375 °C. (*b*) Selected hysteresis curves of the same sample at different temperatures, as indicated by the labels. MOKE was performed in polar geometry, with the polarizers at 45°, using the following acquisition parameters: AC gain = 10×, DC gain = 1×, PEM frequency *f* = 50.037 kHz, lock-in amplifier tuned on 2*f* and number of averaged loops = 5. Temperature dependence of (*c*) the coercive field and (*d*) the Kerr rotation angle at saturation, θ_K,sat_.

The magnetic state of the film was imaged as a function of temperature using XMCD-PEEM at the Co *L*_3_ edge (*h*ν = 778.1 eV). Prior to the experiments, the sample was saturated with an out-of-plane field and then partially demagnetized. To achieve this, an AC field was applied along the surface normal, its amplitude being slowly reduced to zero. In this way, micron-sized domains were nucleated. Fig. 8 ▸(*a*) shows the XMCD-PEEM images acquired at zero field at different temperatures between 25 and 375 °C. The images show a pattern with alternating dark and bright regions, which, in agreement with our previous work, is interpreted as being due to out-of-plane domains with an antiparallel orientation of the magnetization (Genoni *et al.*, 2018 ▸). The XMCD asymmetry is close to 6%, as expected for metallic Co in our measurement geometry (grazing incidence illumination at 16° reduces the contrast by a factor of about 3.5 for the out-of-plane magnetization compared to the in-plane magnetization). Whereas no significant change in domain morphology is observed up to the image at 308 °C, the domain size decreases notably at higher temperatures due to the fragmentation of the domains. The vertical striations affecting all XMCD images are not of magnetic origin. They are caused by the inhomogeneity of the (improperly) focused X-ray-beam spot on the sample, which drifts during the acquisition of the PEEM images with opposite photon helicity.

Even though PEEM is capable of operation under magnetic fields (Sandig *et al.*, 2012 ▸), there are limitations on the maximum field strength that can be applied to the sample. In the specific case of the SPELEEM in Trieste, a special cartridge was developed to apply fields up to 4.5 mT while imaging (Genuzio *et al.*, 2019*b* ▸), but the field application comes at the expense of heating, thus making such a cartridge unsuitable for *in situ* growth experiments. Due to the local nature of the probe, PEEM delivers a qualitative rather than a quantitative description of the magnetization process taking place on the sample. A complementary experimental approach based on MOKE is therefore useful when investigating the behaviour under field.

MOKE hysteresis loops were subsequently measured on another sample with identical magnetic properties, checked by XMCD-PEEM, and similar Co coverage (namely 5.0 ML). Fig. 8 ▸(*b*) shows selected MOKE loops acquired at increasing sample temperatures (from bottom to top), after subtraction of the linear contribution from the UHV windows and Re substrate. Each curve is the average over five consecutive hysteresis loops of 160 data points. The overall acquisition time was 339 s. As can be seen, the graphene/Co sample exhibits a square hysteresis in polar MOKE at room temperature, as is expected for perpendicular magnetic anisotropy, and large coercivity (63 mT). In the RT loop, we obtain a value of 8.0 mdeg for the Kerr rotation, with a signal-to-noise (S/N) ratio slightly less than 48. Considering the Co thickness of about 5 ML of our test sample, we estimate that monolayer sensitivity can be achieved with our instrument at a very decent S/N ratio of about 10. The temperature dependence of the coercive field, H_c_, and Kerr rotation at saturation, θ_K,sat_, are shown in Figs. 8 ▸(*c*) and 8 ▸(*d*), respectively. As can be seen, the coercivity decreases with increasing temperature, following a downward trend when approaching the Curie temperature. The behaviour of the Kerr rotation at saturation suggests that a significant decrease of the magnetization occurs only above 350 °C. The results are consistent with the literature data on similar systems (Ajejas *et al.*, 2018 ▸, 2020 ▸) and will be discussed in more depth in future work.

## 5. Conclusions   

We presented the design, optical layout and performance of a UHV-compatible custom-built MOKE magnetometer, available as a user facility beside the PEEM at the Nanospectroscopy beamline of the Elettra synchrotron. The magnetometer features a liquid-nitrogen-cooled electromagnet producing magnetic fields up to 140 mT at the sample. Both longitudinal and polar measurement geometries can be realized. The MOKE optics employ a photoelastic modulator for conditioning the light beam at high frequencies. A lock-in amplifier is used for detecting the magnetic signal.

The magnetometer is fitted onto a UHV chamber which enables sample preparation with basic surface science tools, *e.g.* gas line and sputter gun. Electron beam evaporators are available for the deposition of metallic and oxide overlayers under well-defined conditions. Sample manipulation is possible thanks to a liquid-nitrogen-cooled rotatable xyz manipulator. The MOKE set-up takes advantage of the full compatibility with the Elmitec PEEM sample manipulation system. A UHV bag enables sample transfer between the two instruments.

The MOKE magnetometer has been tested in realistic surface science experiments on cobalt thin layers, demonstrating a good S/N ratio down to a few layer thicknesses. The experiments on graphene/Co thin films highlighted the benefits of performing combined MOKE and XMCD-PEEM analyses, aiming to correlate the average magnetic behaviour under field to the local properties. Both aspects are key to obtain both a qualitative and quantitative understanding of the magnetic properties, especially when exploring their dependence on controllable parameters, such as temperature, thickness or adsorbate coverage.
